# Advances in Novel Detection Technologies for Occult Hepatitis B Virus Infection: Building an Ultra-Sensitive Barrier for Transfusion Safety

**DOI:** 10.3390/microorganisms13122821

**Published:** 2025-12-11

**Authors:** Meng Yi, Yuwei Hu, Bin Fan, Yiming Pan, Bo Pan, Jue Wang, Zhong Liu

**Affiliations:** 1 Institute of Blood Transfusion, Peking Union Medical College, Chinese Academy of Medical Sciences, Chengdu 610052, China; yimm0317@163.com (M.Y.); yuweihu007@foxmail.com (Y.H.); binguog@sina.com (B.F.); pym031000@163.com (Y.P.); pb199805@163.com (B.P.); wangjue@ibt.pumc.edu.cn (J.W.); 2 Key Laboratory of Transfusion Adverse Reactions, Chinese Academy of Medical Sciences, Chengdu 610052, China; 3 School of Population Medicine and Public Health, Peking Union Medical College, Chinese Academy of Medical Sciences, Beijing 100730, China

**Keywords:** OBI, transfusion safety, blood screening, ultrasensitive detection, ddPCR, CRISPR-Cas

## Abstract

Occult hepatitis B virus infection (OBI), characterized by extremely low viral loads and the persistent intrahepatic presence of cccDNA, poses a profound challenge to global public health security. With a prevalence ranging from 0.06% to over 15% in different donor populations, OBI maintains a risk of transmission and can progress to hepatocellular carcinoma. Its prevention and control have long been limited by the sensitivity constraints of conventional detection methods, highlighting the urgent need for more sensitive diagnostic innovations. Emerging technologies offer distinct breakthroughs: ddPCR facilitates absolute quantification; CRISPR-Cas systems coupled with isothermal amplification enable rapid, point-of-care testing; third-generation sequencing resolves viral integration and mutations; and nanomaterials enhance the signal detection. This review synthesises advancements in OBI diagnostic technologies and provides a comparative overview of their strengths, limitations, and transfusion safety implications, as well as their potential applications in blood transfusion. Recommendations are also proposed to inform the advancement of OBI risk control in blood transfusion and to guide the development of novel diagnostic technologies, particularly relevant to regions with high HBV endemicity, such as China.

## 1. Introduction

Despite more than three decades of clinical availability of the HBV vaccine, HBV infection remains a major worldwide public health challenge [1]. Epidemiological data show that approximately one quarter of chronic HBV carriers progress to cirrhosis or hepatocellular carcinoma (HCC), which together account for up to 820,000 deaths annually, with the burden particularly high in African regions and the Western Pacific [2,3]. Notably, in individuals who are seronegative for hepatitis B surface antigen (HBsAg), replication-competent HBV DNA can still be detected in the liver and blood of some carriers, a condition defined as occult hepatitis B virus infection (OBI) [4,5]. OBI is categorized as seropositive (anti-HBc and/or anti-HBs positive) or seronegative (both negative) [6]. Due to the limited sensitivity of conventional serological assays, seronegative occult hepatitis B infection (OBI) cases typically lack overt clinical manifestations and are challenging to detect routinely and timely. It is important to distinguish OBI from window period infections, where HBsAg is negative but HBV DNA is detectable during early acute infection. Unlike window period infections, OBI represents a chronic state with persistent cccDNA, which can reactivate under immunosuppression [7]. Studies demonstrate that HBV DNA in the peripheral blood of patients with occult hepatitis B infection (OBI) typically exhibits intermittent low-level viremia, generally below the detection threshold of 200 IU/mL (i.e., ≈1000 copies/mL) [8]. The core mechanism underlying OBI is the persistent latency of cccDNA in hepatocyte nuclei [9]. cccDNA serves as a transcriptional template for viral replication, but its low-level transcription and immune control lead to intermittent viremia, with viral loads fluctuating below conventional detection limits [7].

Low-level viremia and the limited sensitivity of current detection technologies markedly increase the transmission risk of OBI. The sensitivity threshold of existing nucleic acid amplification testing (NAT) is 0.15 IU/mL [10], whereas the viral load of OBI often falls below this threshold. Moreover, the Poisson distribution characteristics of viral nucleic acids increase the likelihood of false negatives in pooled-sample testing, with the miss-detection rate reaching 29% in six-sample pools [11]. Epidemiological studies have shown that the positive rate of HBV DNA among HBsAg-negative blood donors ranges from 0.094% to 0.5%, with anti-HBc–only positive individuals carrying a higher risk of transmission [12,13,14]. Transfusion transmission can not only result in lifelong infection in recipients but also trigger viral reactivation under immunosuppressive conditions (with reactivation rates reaching 20–50% in patients with hematologic malignancies), and may even lead to fulminant hepatitis [15,16]. Notably, OBI prevalence may vary between first-time and repeat blood donors. Studies suggest that repeat donors have a lower OBI risk due to prior screening, highlighting the need for tailored screening strategies [12,14].

The current prevention and control system faces dual technical bottlenecks: conventional serology combined with NAT lacks sufficient sensitivity for low-load samples, and it is unable to effectively distinguish cccDNA from extracellular hepatitis B virus DNA (relaxed circular DNA, rcDNA) [17]. Meanwhile, pooled testing inevitably results in a high rate of false negatives. Given these limitations, developing ultra-sensitive assays has become a priority. This review summarizes the latest advances in novel pathogen detection strategies related to OBI, aiming to overcome the issue of “low-load missed detection” and to establish a safer safeguard for blood transfusion. Integrating novel technologies like ddPCR or CRISPR-Cas into current NAT workflows (e.g., MP-NAT or ID-NAT) requires consideration of cost, throughput, and infrastructure. For instance, CRISPR-Cas assays could be used as a rapid pre-screening tool before NAT, while ddPCR may serve as a confirmatory test for low-load samples [18,19].

## 2. Novel OBI Detection Technologies

Current HBV DNA detection in practice is largely NAT-based [20], including sequencing [21], nested polymerase chain reaction (PCR) [22], fluorescent quantitative polymerase chain reaction (qPCR) [23] and Southern blotting [24], all of which have significantly enhanced blood donation safety. In China, HBV DNA detection is typically performed using qPCR [25]. Due to the high cost and time-consuming nature of individual donor NAT (ID-NAT), most blood centers commonly employ mini pool NAT (MP-NAT) for HBV screening [19]. However, the HBV DNA levels in OBI donors are sometimes below the detection limit (LOD) of MP-NAT, which may result in a small proportion of OBI cases being missed [8]. The working principle of qPCR is based on the detection and quantification of fluorescent reporter molecules; however, amplification efficiency is influenced by multiple factors and cannot remain constant throughout the reaction, which can compromise the accuracy of quantification [26]. Therefore, it is necessary to identify a more sensitive, specific, and reliable method to enhance the detection rate of OBI and maximize the safety of blood transfusion.

### 2.1. dPCR and ddPCR

Digital polymerase chain reaction (dPCR), as a third-generation PCR technology, originates from the combination of conventional PCR amplification techniques and microfluidic technology [27]. This technique relies on limiting dilution, end-point PCR amplification, and Poisson distribution statistics, with its core principle being the absolute quantification of nucleic acid molecules through end-point measurement. In practice, dPCR first partitions the reaction mixture into thousands to millions of individual micro-reaction units, ensuring that each unit contains zero, one, or very few copies of the target nucleic acid. These partitions are then subjected to PCR amplification until the endpoint is reached. Finally, a fluorescence detection system is used to identify positive and negative partitions, and the absolute copy number is computed from the occupancy parameter (λ) of a Poisson model, with the accuracy expressed as statistical uncertainty (e.g., 95% CI). This partitioning approach markedly enhances detection sensitivity and, by concentrating target sequences within isolated micro-reactors, effectively reduces competition between templates, thereby enabling efficient detection of rare mutations against a high-abundance wild-type sequence background [28]. In 2011, Hindson and colleagues further innovated on conventional dPCR by developing droplet digital polymerase chain reaction (ddPCR) [29,30], which allows partitioning the sample into thousands of nanoliter-scale droplets, enabling PCR to run separately in every droplet.

Research on ddPCR technology in the field of HBV DNA detection has been increasingly in-depth. Its potential as an auxiliary diagnostic tool for OBI has been preliminarily validated, yet its widespread clinical adoption still faces challenges. Through quantitative analysis of serum HBV DNA that ddPCR results, Piermatteo et al. [31] demonstrated that ddPCR showed a strong linear concordance with qPCR (R^2^ > 0.98) and a ≈10-fold lower limit of detection (1.5 vs. 15 IU/mL). Moreover, validation across multiple concentration gradients confirmed that ddPCR provides accurate, reproducible, and highly sensitive quantification ability of serum HBV DNA, supporting the method’s stability and reliability. ddPCR technology exhibits excellent resistance to interference and precise quantification capabilities. The Hayashi team [23] successfully achieved specific quantification of HBV cccDNA using ddPCR, consistently detecting ≥5 copies of cccDNA templates even in the presence of a 10^3^-fold excess of linear HBV DNA interference. Given that serum HBV RNA levels can objectively reflect the transcriptional activity of intrahepatic HBV cccDNA, Limothai et al. [32] demonstrated that ddPCR increased the detection rate of HBV RNA in low-viral-load patients (<500 IU/mL) by more than threefold compared to qPCR, achieving 97% accuracy for detecting 5 copies per reaction and significantly enhancing the analytical performance for extremely low-abundance nucleic acid specimens. Taken together, these findings indicate that ddPCR provides clear technical advantages for detecting low-level pathogens such as OBI.

A comparison of ddPCR with currently widely used detection methods is shown in Table 1. Although ddPCR has been shown to offer significant advantages over qPCR, its high cost and low throughput limit its broader adoption in clinical practice. To address these issues, the Wang team [27] developed a novel modular ddPCR system that, by simplifying the microfluidic chip design and employing an open fluorescence detection module, substantially improves cost-effectiveness while maintaining the key performance of commercial equipment. Prospective studies [33] indicate that next-generation technologies will enable simultaneous multi-target detection within a single tube through the integration of multi-color fluorescence channels, thereby enhancing multi-parameter analytical capability compared to current systems. These breakthrough advances collectively indicate a promising potential for ddPCR in high-sensitivity detection scenarios, such as OBI screening among blood donors. It is important to note that the sensitivity of ddPCR for OBI detection can be influenced by pre-analytical factors, such as sample volume and DNA extraction efficiency. Inadequate extraction may reduce the yield of low-abundance HBV DNA, leading to false negatives. Standardizing these factors is crucial for reliable ddPCR application in clinical settings [31,32].

### 2.2. CRISPR-Cas System

In recent years, the CRISPR-associated (Cas) nuclease system, derived from clustered regularly interspaced short palindromic repeats (CRISPR), has received increasing attention. This system originates from the adaptive immune mechanism of prokaryotes and was first discovered by Ishino et al. in *Escherichia coli* in 1987 [37]. In the field of pathogen diagnostics, a key turning point occurred in 2016 with the discovery of the trans-cleavage activity of Cas12/Cas13, namely, the non-specific degradation of reporter molecules following target recognition. Compared with other rapid detection techniques, such as recombinase polymerase amplification (RPA) and loop-mediated isothermal amplification (LAMP), CRISPR/Cas-based assays exhibit higher sensitivity and specificity [38]. Since the advent of CRISPR-Cas detection technology, scientists have developed multiple detection platforms based on this system, including SHERLOCK (Cas13a), DETECTR (Cas12a), CDetection (Cas12b), and Cas14-DETECTR. These platforms support quick testing with markedly improved sensitivity and precise identification for a wide range of pathogens [39,40].

**Figure 1:**
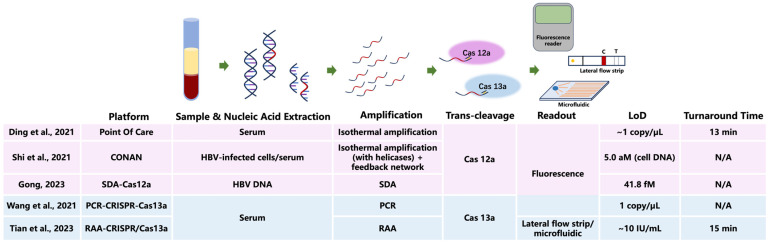
Workflow diagram of CRISPR-Cas12a/Cas13a assays for low-level HBV DNA detection and comparison of representative platforms. Limit of detection (LoD) values are reported as in the original publications (copy/μL, aM, fM, IU/mL) [18,41,42,43,44]. N/A, not available. CRISPR, clustered regularly interspaced short palindromic repeats; Cas, CRISPR-associated protein; HBV, hepatitis B virus; SDA, strand displacement amplification; PCR, polymerase chain reaction; RAA, recombinase-aided amplification; IU, international unit; aM, attomolar; fM, femtomolar.

CRISPR-Cas detection technology has been extensively investigated in low-load HBV detection (Figure 1). The Ding team [18] investigated the potential application of rapid on-site detection technology based on CRISPR-Cas12a for HBV. This technology can detect HBV DNA at concentrations as low as 1 copy/μL within 13 min, demonstrating high sensitivity and specificity, while being cost-effective and suitable for on-site testing in resource-limited settings. With a detection limit near 1 copy·μL^−1^ and results available in 13 min, this technology combines high sensitivity and specificity with favorable cost, allowing on-site testing in settings where resources are limited. The Shi team [41] developed a CRISPR-Cas12a self-catalyzed, feedback-driven nucleic acid circuit system (CRISPR-Cas-only amplification network, CONAN). This system utilizes the collateral cleavage activity of Cas12a, which non-specifically degrades fluorescent reporter molecules (e.g., FAM-labeled ssDNA) upon target recognition, generating a fluorescence signal. Unlike traditional PCR, which relies on thermal cycling and DNA polymerization, CONAN achieves exponential amplification isothermally through a feedback loop, without the need for enzymes like polymerases. Results indicate that the CONAN system can detect genomic DNA in HBV-infected cells at concentrations as low as 5.0 aM and can also be applied to clinical serum samples from both HBV-infected and uninfected individuals, yielding results consistent with those obtained by qRT-PCR. Similarly, a team [42] combined strand displacement amplification (SDA) technology with CRISPR-Cas12a to detect low-load HBV markers. This approach enables highly sensitive detection of HBV DNA, with a detection limit as low as 41.8 fM. For perspective, 5.0 aM corresponds to approximately 3 copies/μL, and 41.8 fM is about 25,000 copies/μL, whereas typical qPCR assays for HBV have a detection limit of 10–100 copies/mL [23,26]. Furthermore, this approach can also discriminate DNA sequences with base mutations, demonstrating excellent detection specificity. The Wang team [43] developed a PCR-CRISPR-HBV DNA detection system based on CRISPR-Cas13a, capable of detecting HBV DNA at 1 copy/μL. In a study of 312 serum samples from HBV patients, the system effectively identified 10 low-copy samples that were negative by qRT-PCR. The Tian team [44] developed a RAA-CRISPR/Cas13a-based lateral flow strip assay that permits the detection of HBV at concentrations down to ~10 IU/mL. Using colloidal gold strips or microfluidic chips enables “sample-in, result-out” detection within 15 min, at a cost of less than $1 per sample, making it suitable for integration into rapid screening scenarios such as blood donation vehicles. Despite remaining challenges such as gRNA off-target effects and interference from complex sample inhibitors, CRISPR-Cas technology, with its ultra-sensitivity, portability, and low cost, holds promise to become the next-generation gold standard for OBI screening in blood donors.

### 2.3. Third-Generation Sequencing Technologies

With the development of third-generation sequencing (TGS) technologies, particularly single-molecule real-time sequencing platforms such as Oxford Nanopore, it is now possible to detect HBV integration sites across the whole genome, as well as structural variations including chromosomal translocations and gene fusions. TGS thus serves as a powerful tool for comprehensively studying HBV integration within the host genome. These technologies provide new perspectives for understanding the genetic complexity of HBV and for detecting viral infections. With single-molecule real-time sequencing (e.g., Nanopore or PacBio platforms), TGS reaches a limit of detection of 1–5 copies per mL, roughly 10–100 times lower than qPCR [45]. McNaughton et al. [46] developed a circular-genome-targeted approach in which isothermal RCA enriches HBV DNA and generates long concatemers containing contiguous copies of the genome. Sequencing these products via TGS increased HBV sequencing accuracy to 99.7% and enabled the resolution of intrahost sequence variants. Integrating isothermal amplification and TGS could enable rapid, real-time detection of HBV. Lee et al. [47] developed a TGS approach based on multiplex PCR to obtain the full genome of HAV, a method that can also be applied to HBV genome analysis. TGS has shown that, for viral nucleic acid loads between 10 and 10^5^ copies per μL, genome coverage can reach 90.4% to 99.5% within 8 h.

With its ultra-sensitivity, integrative resolution, and high-throughput adaptability, TGS is expanding the diagnostic paradigm for HBV and OBI screening. However, TGS faces challenges such as gRNA off-target effects. In gRNA-based enrichment methods, guide RNAs are designed to target specific HBV sequences for amplification or capture before sequencing. Off-target binding can lead to false positives or reduced sensitivity. Optimizing gRNA design and using computational tools can mitigate this issue [46,47]. In the context of OBI screening in blood donation samples, the integration of TGS with high-fidelity CRISPR-Cas12a technology, lyophilized reagent development, and artificial intelligence (AI)-based automated interpretation could bring transformative improvements.

### 2.4. Nanomaterial-Based Detection Technologies

Nanomaterials, owing to their characteristic mechanical, electronic, and optical traits, enable highly sensitive HBV detection by either amplifying the detection signal or specifically recognizing target molecules. For signal amplification, the surface plasmon resonance (SPR) of gold nanoparticles (AuNPs) can lead to a red→blue shift in coloration, allowing visual detection of HBsAg at concentrations as low as 0.1 ng/mL [48]. Nanomaterials like graphene and carbon nanotubes (CNTs) can be applied to modify electrodes, increasing the specific surface area, accelerating electron transfer, and enhancing current or impedance signals. Based on this principle, HBV DNA detection can be achieved at concentrations as low as 111 copies/mL [49]. Quantum dots (QDs) exhibit high-intensity, stable fluorescence upon excitation, and their use in fluorescence immunoassays for HBsAg detection can lower the detection limit to 1.16 pg/mL [50].

Magnetic nanoparticles (MNPs), owing to their unique physicochemical properties, have become a key tool for enhancing diagnostic sensitivity and efficiency in HBV detection. MNPs (with diameters of 1–100 nm) can be functionalized to present HBV-targeting antibodies or aptamers, enabling the capture of HBV antigens (such as HBsAg) or viral particles from blood through antigen–antibody interactions. Upon application of an external magnetic field, MNP–target complexes can be rapidly separated, concentrating low-abundance pathogens and reducing background interference. MNPs conjugated with anti-HBs antibodies can specifically capture HBsAg from serum at concentrations as low as 5 IU/mL [51]. Moreover, due to their abundant active sites and larger catalytic surface area, MNPs can be combined with various nanomaterials to produce synergistic effects. For example, magnetite (Fe_3_O_4_) MNPs, serving as efficient biocarriers, can function as probe capture agents, while silica nanoparticles (SiNPs) loaded with rhodamine B act as fluorescent capsules and signal amplifiers, sequestering the MNPs and liberating the encapsulated dye. In this system, fluorescence intensity exhibits a linear correlation with HBsAg concentrations over the range 6.1 ag/mL to 0.012 ng/mL, with the minimum detectable limit of 5.7 ag/mL [52]. Sea urchin-like dual MNPs, gold–platinum hybrid MNPs, and L-cysteine-bridged gold–silver MNPs are currently among the most widely developed signal-generating molecules. Three-dimensional SnO_2_-loaded graphene sheets (GS-SnO_2_-BMNPs) can be integrated with these MNPs to build a sandwich immunosensor for the simultaneous detection of HBsAg and HBeAg. Based on these platforms, Dong and colleagues [53] constructed a sandwich-type electrochemical immunosensor based on MoO_2_ nanosheets (MoO_2_ NSs) decorated with gold-core, palladium-shell nanodendrites (Au@Pd NDs) for HBsAg detection, reaching a limit of detection of 3.3 fg/mL. For further investigations into the application of nanomaterials in low-load HBV detection, see Table 2. Despite the high sensitivity, nanomaterial-based assays face challenges in clinical translation. For example, whole blood components can cause interference, leading to false signals. Additionally, the stability and batch-to-batch variability of nanomaterials require rigorous standardization. Future efforts should focus on integrating nanomaterials with microfluidic chips to minimize interference and improve robustness [54].

Compared with traditional methods, nanomaterials have attracted considerable attention in primary blood collection institutions for OBI screening due to their high sensitivity, short detection time, and minimal equipment and personnel requirements. However, the currently developed nanomaterials are generally expensive, cannot fully eliminate interference from whole blood, and have yet to be produced at a large-scale standardized level. The application of nanomaterials in OBI screening still has a long way to go. Future development of integrated microfluidic chips combined with AI-based intelligent analysis could amplify the advantages of nanoplatforms—“specific recognition, signal amplification, and portable output”—providing innovative tools for OBI screening at the primary care level.

### 2.5. Comparative Analysis and Practical Readiness for Transfusion Screening

To provide a clearer sense of real-world readiness, we qualitatively compared the four major technology families discussed above—ddPCR, CRISPR-Cas-based assays, TGS, and nanomaterial-based platforms—in terms of analytical performance and implementation characteristics (Table 3).

## 3. Summary and Outlook

Building on the comparative assessment above, future OBI prevention and control should focus on technological integration and clinical translation. In the field of detection, the high cost and low throughput of ddPCR can be mitigated through modular chip design and the integration of multi-color fluorescence channels, promoting its large-scale application in blood donation screening. CRISPR-Cas systems need to overcome challenges such as gRNA off-target effects and interference from complex samples, while combining with lyophilized reagent development to enhance the stability of on-site testing. TGS technology should be integrated with artificial intelligence to enable automated analysis of large datasets, thereby improving the clinical interpretability of HBV integration sequences. Future directions include the use of AI-driven algorithms to integrate multi-parameter data (e.g., from ddPCR, CRISPR, and antigen markers) for improved OBI detection. Machine learning models can enhance interpretation accuracy and reduce false positives. Research on inactivation technologies must balance pathogen clearance efficacy with the functional preservation of blood components, while exploring novel photochemical or nanomaterial-based strategies targeting cccDNA. For clinical translation, key milestones include regulatory validation (e.g., FDA approval), multi-center clinical trials to establish sensitivity and specificity, and cost-effectiveness analyses. For example, ddPCR requires validation in large donor cohorts to standardize its use in blood screening. Furthermore, harmonization of OBI testing standards globally, especially in high-endemicity regions like China, is essential to ensure transfusion safety. International collaborations should establish guidelines for technology adoption and quality control. Moreover, multi-platform integration is an inevitable trend. For example, CRISPR-based pre-enrichment combined with ddPCR absolute quantification can establish a two-tiered “screening–confirmation” system, while nanomaterial-based microfluidic chips integrated with AI analysis have the potential to achieve an all-in-one “recognition–signal amplification–output” workflow. These breakthroughs will drive OBI detection from a “single-target” approach toward “multi-dimensional markers (DNA/RNA/antigen),” ultimately establishing an ultra-sensitive, low-cost, fully automated blood safety barrier and providing critical technological support for global hepatitis elimination initiatives.

## Figures and Tables

**Table 1:** Comparison of Common HBV Diagnostic Technologies and Their Application Scenarios.

Technique	ELISA	NAT	ddPCR
Target	Antigen or antibody	Nucleic acid	Nucleic acid
Principle	Antigen–antibody reaction	Nucleic acid amplification (e.g., PCR, RT-PCR) is used to detect the DNA or RNA of pathogens.	By distributing the sample across droplets/microchambers, performing independent PCR with fluorescence readout, and applying Poisson-based quantification.
Sensitivity	pg/mL	The detection rate of HBV nucleic acid is 100% [34]	Ultra-low-frequency calling of rare mutations or single-copy molecules (≈0.001%) [35]
Specificity	Low specificity and potential cross-reactivity	High	High
Detection Time	1.5–4 h	1.5–2.5 h	4–6 h
Throughput	High	Medium	Low [36]
Cost	Low	Medium	High
Application Scenario	Mass screening Resource-limited settings	Specialized laboratory	Specialized laboratory
OBI Screening	Combined testing (HBsAg + anti-HBc + anti-HBs) is used for initial screening in resource-limited settings, but it is limited by the serological window period.	Gold standard for diagnosis	No standard curve is required; it allows accurate calculation of viral copy numbers and is currently suitable for research applications, enabling precise monitoring of OBI viral loads.

ELISA: Enzyme-Linked Immunosorbent Assay; NAT: Nucleic Acid Testing; ddPCR: Droplet Digital PCR.

**Table 2:** Applications of Nanomaterial-Based Detection for HBV and HCV.

Structure	Nanomaterial	Target	Detection Method	Detection Limit	Linear Range	Ref
Nanoparticles	Anti-HBs and HAT-coated AuNPs	HBsAg	Fluorescent ELISA	5 × 10^−4^ IU/mL	None	[48]
	AuNPs	HBsAg	Color change in solution caused by surface plasmon resonance (SPR)	0.1 ng/mL (visual (by naked eye)) 0.01 ng/mL (instrument-Based)	0.01–10 ng/mL	[55]
	AuNPs	HBV DNA	ELISA	111 copies/mL	10^2^–10^5^ copies/mL	[49]
	Bifunctionalized AuNPs	HBV DNA	Chemiluminescence	5.9 × 10^−12^ M	None	[56]
	Au@Fe_3_O_4_@SiO_2_ NPs	HBsAg	Chemiluminescence	0.05 ng/mL	1–225 ng/mL	[57]
	AuNPs	HBV DNA	Lateral flow immunochromatography	10^3^ copies/mL	None	[58]
	AuNRs	HBsAg	Surface plasmon resonance (SPR)	0.1 IU/mL	0.01–1 IU/mL	[59]
	AuNRs	HBV DNA	Fluorescence resonance energy transfer (FRET)	15 pmol/L	0.045–6.0 nmol/L	[60]
	MNPs	HBsAg	Magnetically assisted fluorescence immunoassay	5 IU/mL	None	[51]
	Silica NPs	HBV DNA	Resonant frequency shift	2.3 × 10^−15^ M	23.1 fM–2.3 nM	[61]
QDs	QDs Nanobeads	HBsAg	Immunofluorescence	0.078 ng	None	[62]
	QDs	HBV DNA	Fluorescence resonance energy transfer (FRET)	1.5 nmol/L	2.5–30 nmol/L	[63]
	Graphene Quantum Dots (Graphene QDs)	HBV DNA	Differential pulse voltammetry (DPV)	1 nM	10–500 nM	[64]
One-Dimensional (1D) Material	Silicon Nanowires	HBsAg and HBx	Electrochemical	100 fg/mL	None	[65]
	Silicon Nanowires	HBV DNA	Fluorescence quenching	20 copies per reaction	None	[66]
	Amino-Functionalized Carbon Nanotubes	HBcAb	Square-wave voltammetry (SWV)	0.03 ng/mL	0.03–6 ng/mL	[67]
Two-Dimensional (2D) Material	Pencil Graphite Electrode	HBV DNA	ELISA	2.48 µg/mL	5–30 µg/mL	[68]
Nanocomposite Materials	Fe_3_O_4_ MNP and AuNPs	HBsAg	Square-wave voltammetry (SWV)	0.19 ng/µL	0.3–1000 ng/µL	[69]
	GO-GNRs	HBsAg	Surface-enhanced spectroscopy	0.05 pg/mL	1–1000 pg/mL	[70]
	Au@Pd Nanodendrites/ NH_2_-MoO_2_ Nanosheets	HBsAg	Electrochemical	3.3 fg/mL	10–100 ng/mL	[53]

HBsAg: Hepatitis B Virus Surface Antigen; HAT: Human α-Thrombin; ELISA: Enzyme-Linked Immunosorbent Assay; AuNPs: Gold Nanoparticles; MNPs: Magnetic Nanoparticles; Silica NPs: Silica Nanoparticles; QDs: Quantum Dots.

**Table 3:** Comparative overview of emerging HBV/OBI detection technologies.

Technology	Analytical LoD Level	Throughput (per Run)	Per-Test Cost	Sample Requirements	Operational Complexity	Suitability for Blood Centers
ddPCR	~1–10 IU/mL-level	Low [36]	High	Extracted plasma/serum DNA	High—requires specialized equipment and trained personnel	High
CRISPR-Cas system	Single-copy/µL or aM–fM	Low–medium	Low	Extracted plasma/serum DNA with isothermal amplification	Moderate—multi-step workflows but amenable to integration into cartridges	High
TGS	~10^2^–10^3^ copies/mL	High	High	Extracted plasma/serum DNA with complex library preparation	Very high—requires specialized platforms and bioinformatics	Low
Nanomaterial-based assays	Antigen: fg–pg/mL DNA: fM–pM or ~10^2^–10^3^ copies/mL	Low–medium	High	Serum/plasma	Moderate—often requires careful surface chemistry and calibration	High

## Data Availability

No new data were created or analyzed in this study. Data sharing is not applicable to this article.

## Abbreviations

The following abbreviations are used in this manuscript:

HBV	Hepatitis B virus
HCC	Hepatocellular carcinoma
OBI	Occult hepatitis B virus infection
HBsAg	Hepatitis B surface antigen
anti-HBc	Hepatitis B core antibody
anti-HBs	Hepatitis B surface antibody
cccDNA	Covalently closed circular DNA
rcDNA	Relaxed circular DNA
NAT	Nucleic acid testing
PCR	Polymerase chain reaction
qPCR	Quantitative polymerase chain reaction
dPCR	Digital polymerase chain reaction
ddPCR	Droplet digital polymerase chain reaction
LoD	Limit of detection
RPA	Recombinase polymerase amplification
LAMP	Loop-mediated isothermal amplification
CRISPR	Clustered regularly interspaced short palindromic repeats
Cas	CRISPR-associated
SHERLOCK	Specific High-sensitivity Enzymatic Reporter unLOCKing
DETECTR	DNA Endonuclease-Targeted CRISPR Trans Reporter
SDA	Strand displacement amplification
qRT-PCR	Quantitative reverse transcription polymerase chain reaction
TGS	Third-generation sequencing
RCA	Rolling-circle amplification
ELISA	Enzyme-linked immunosorbent assay
